# The complete chloroplast genome sequence of Japanese buttercup *Ranunculus japonicus* Thunb.

**DOI:** 10.1080/23802359.2021.1987166

**Published:** 2021-10-14

**Authors:** Wen-Qiong Zeng, Hua-Jian Yan, Yun-Zhe Wu, Rui-rui Wang, Xian-Fei Xiao, Zhe-Chen Qi, Xiao-Ling Yan

**Affiliations:** a Zhejiang Province Key Laboratory of Plant Secondary Metabolism and Regulation, College of Life Sciences and Medicine, Zhejiang Sci-Tech University, Hangzhou, China; b Shaoxing Academy of Biomedicine of Zhejiang Sci-Tech University, Shaoxing, China; c Agricultural and Rural Development Service Center of Chun’an County, Hangzhou, China; d Shanghai Chenshan Plant Science Research Centre, Chinese Academy of Sciences, Shanghai Chenshan Botanical Garden, Shanghai, China

**Keywords:** *Ranunculus japonicus*, Ranunculaceae, chloroplast genome, phylogenomic analysis

## Abstract

*Ranunculus japonicus* is an important medicinal herb widely used in East Asia. In this study, we report the first complete chloroplast genome sequence of *Ranunculus japonicus* using next-generation sequencing technology. The chloroplast genome size of *R. japonicus* was 156,981 bp. A total of 129 genes were included, consisting 84 protein-coding genes, eight rRNA genes, and 37 tRNA genes. Thirteen protein-coding genes had intron (*ycf3* gene, *rps12* gene, *rps12* gene, *clpP* gene contained two introns). A further phylogenomic analysis of Ranunculaceae, including 10 taxa, was conducted for assessing the placement of *R. japonicus*. It will provide valuable genetic information for this medicinally important species.

*Ranunculus japonicus* Thunberg 1794 is a topical herb widely distributed in East Asia (Cao et al. 1992). It inhabits the moist grasslands and mountains. As a traditional Chinese medicine, it is widely used to treat various diseases, including malaria, jaundice, migraines and stomachaches for over 1800 years (Rui et al. 2010). In Korea, the young stems of *R. japonicus* are eaten as a vegetable (Yun et al. 2021). In addition, recent study showed that 27 validated compounds of *R. japonicus* were predicted to exhibit therapeutic effects against rheumatoid arthritis, which offered a potential clinical application (Wang et al. 2021). However, there are few relevant reports about the genetic information and phylogenetic relationships of *R. japonicus* and its relatives. In the study, the first complete chloroplast genome of *R. japonicus* was assembled and characterized. It will provide potential genetic resources for further population genetic study of this medicinally important species.

The *R. japonicus* individual was collected from Xuancheng, Anhuhi, China (GPS: 118°41′30.73″, N 30°34′15.49″). DNA was extracted from its silica dried leaves using DNA Plantzol Reagent (Invitrogen, Carlsbad, CA, USA) in accordance with the manufacturer’s instructions. The specimen and extracted DNA was deposited at Zhejiang Province Key Laboratory of Plant Secondary Metabolism and Regulation, Zhejiang Sci-Tech University (http://sky.zstu.edu.cn) under the voucher number ZSTU00034 (collected by Zhe-Chen Qi and zqi@zstu.edu.cn). Sequencing libraries were prepared using Illumina’s TruSeq Nano DNA Library preparation kit (350 bp median insert) following the manufacturer’s protocol. The plastome sequences were generated using the Illumina HiSeq 2500 platform (Illumina Inc., San Diego, CA, USA). In total, about 14.7 million high-quality clean reads (150 bp PE read length) were generated with adaptors trimmed. These clean data were *de novo* assembled to complete chloroplast genome using GetOrganelle (Jin et al. 2020). Geneious v11.1.5 (Biomatters Ltd, Auckland, New Zealand) was used to annotate the genome with *R. sceleratus* plastome (GenBank: MK253452.1) as a reference.

The full length of the complete *R. japonicus* chloroplast sequence (GenBank Accession No. MZ169045) is 156,981 bp, consisting of a large single copy region (LSC with 85,454 bp), a small single copy region (SSC with 18,897 bp), and two inverted repeat regions (IR with 26,315 bp). The overall GC content of *R. japonicus* chloroplast genome was 37.7%. A total of 129 genes were included in the genome (84 protein-coding genes, 8 rRNA genes, and 37 tRNA genes). Seventeen genes had two copies, which were comprised of 6 PCG genes (*ndhB*, *rps7*, *rps12*, *ycf2*, *rpl2*, *rpl23*), 7 tRNA genes (*trnl-CAU*, *trnl-CAA*, *trnv-GAC*, *trnl-GAU*, *trna-UGC*, *trnR-ACG*, *trnN-GUU*), and all 4 rRNA species (*rrn16*, *rrn23*, *rrn4.5*, *rrn5*). In the genome, nine protein-coding genes (*rps16*, *atpF*, *rpoC1*, *petB*, *petD*, *rpl16*, *rpl2*, *ndhB*, *ndhA*) had one intron, and *ycf3* gene, *rps12* gene, *rps12* gene, *clpP* gene contained two introns.

Ten species with available chloroplast genomes were selected to study the phylogenetic placement of *R. japonicus* in Ranunculaceae. *Caltha palustris* was used as outgroup for constructing the phylogenetic tree. Alignment of plastomes was generated by MAFFT v7.475 (Katoh and Standley 2013). A maximum-likelihood tree was generated by IQTREE v1.6.8 (Nguyen et al. 2015), with the best selected TVM + F+R2 model and 5000 bootstrap replicates. The result showed that *R. japonicus* was closely related to a clade formed by *R. occidentalis,* and *R. austro-oreganus* according the current sampling extent (Figure 1).

**Figure 1.**
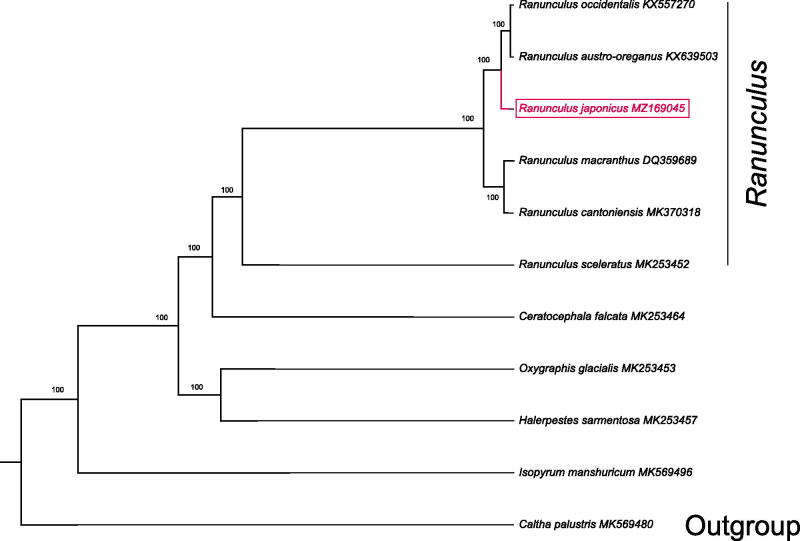
The phylogenetic tree based on 10 complete chloroplast genome sequences in Ranunculaceae (bootstrap values were showed on each node. Accession numbers were listed with each species).

## Data Availability

The associated BioProject, SRA, and Bio-Sample numbers are SUB9615995, PRJNA729222, and SAMN19114502 respectively. The DNA matrix and phylogenetic tree that support the findings of this study are openly available in figshare at https://doi.org/10.6084/m9.figshare.16585595.v1.
